# *In silico* clinical trial evaluating lisdexamfetamine’s and methylphenidate’s mechanism of action computational models in an attention-deficit/hyperactivity disorder virtual patients’ population

**DOI:** 10.3389/fpsyt.2023.939650

**Published:** 2023-06-02

**Authors:** José Ramón Gutiérrez-Casares, Javier Quintero, Cristina Segú-Vergés, Pilar Rodríguez Monterde, Tamara Pozo-Rubio, Mireia Coma, Carmen Montoto

**Affiliations:** ^1^Unidad Ambulatoria de Psiquiatría y Salud Mental de la Infancia, Niñez y Adolescencia, Hospital Perpetuo Socorro, Badajoz, Spain; ^2^Servicio de Psiquiatría, Hospital Universitario Infanta Leonor, Universidad Complutense, Madrid, Spain; ^3^Anaxomics Biotech, Barcelona, Spain; ^4^Structural Bioinformatics Group, Research Programme on Biomedical Informatics, Department of Medicine and Life Sciences, Universitat Pompeu Fabra, Barcelona, Spain; ^5^Medical Department, Takeda Farmacéutica España, Madrid, Spain

**Keywords:** attention-deficit/hyperactivity disorder, lisdexamfetamine, methylphenidate, mathematical modeling, *in silico* clinical trial

## Abstract

**Introduction:**

Attention-deficit/hyperactivity disorder (ADHD) is an impairing psychiatric condition with the stimulants, lisdexamfetamine (LDX), and methylphenidate (MPH), as the first lines pharmacological treatment.

**Methods:**

Herein, we applied a novel *in silico* method to evaluate virtual LDX (vLDX) and vMPH as treatments for ADHD applying quantitative systems pharmacology (QSP) models. The objectives were to evaluate the model’s output, considering the model characteristics and the information used to build them, to compare both virtual drugs’ efficacy mechanisms, and to assess how demographic (age, body mass index, and sex) and clinical characteristics may affect vLDX’s and vMPH’s relative efficacies.

**Results and Discussion:**

We molecularly characterized the drugs and pathologies based on a bibliographic search, and generated virtual populations of adults and children-adolescents totaling 2,600 individuals. For each virtual patient and virtual drug, we created physiologically based pharmacokinetic and QSP models applying the systems biology-based Therapeutic Performance Mapping System technology. The resulting models’ predicted protein activity indicated that both virtual drugs modulated ADHD through similar mechanisms, albeit with some differences. vMPH induced several general synaptic, neurotransmitter, and nerve impulse-related processes, whereas vLDX seemed to modulate neural processes more specific to ADHD, such as GABAergic inhibitory synapses and regulation of the reward system. While both drugs’ models were linked to an effect over neuroinflammation and altered neural viability, vLDX had a significant impact on neurotransmitter imbalance and vMPH on circadian system deregulation. Among demographic characteristics, age and body mass index affected the efficacy of both virtual treatments, although the effect was more marked for vLDX. Regarding comorbidities, only depression negatively impacted both virtual drugs’ efficacy mechanisms and, while that of vLDX were more affected by the co-treatment of tic disorders, the efficacy mechanisms of vMPH were disturbed by wide-spectrum psychiatric drugs. Our *in silico* results suggested that both drugs could have similar efficacy mechanisms as ADHD treatment in adult and pediatric populations and allowed raising hypotheses for their differential impact in specific patient groups, although these results require prospective validation for clinical translatability.

## 1. Introduction

Attention-deficit/hyperactivity disorder (ADHD) is a psychiatric condition well recognized in the pediatric population in which children suffer from persistent inattentiveness, hyperactivity, impulsivity, or any combination thereof (1). ADHD global prevalence in school-age children is estimated at around 7.2% (2) and is generally accepted to range from 6 to 10% (3). This disorder often persists into adulthood, although its presentation can differ since some symptoms may resolve while others endure (3, 4). Notably, comorbid psychiatric disorders are present in up to 67% of ADHD pediatric-adolescent patients (5) and almost 80% of adults (6–13). ADHD patients can be treated with either non-pharmacologic or pharmacologic approaches or combining both strategies (14). Drugs to treat ADHD include, but are not limited to, stimulants [e.g., lisdexamfetamine (LDX) and methylphenidate (MPH)], some of which have extended-release formulations available [e.g., osmotic release oral system (OROS) for MPH].

Methylphenidate is recommended as first-line treatment in children and adolescents by several ADHD management guidelines (15, 16). However, LDX has been found to be superior to OROS-MPH in some studies (17–19), although these have mainly been performed with indirect designs, such as systematic reviews or *post hoc* analyzes. Two head-to-head studies have compared LDX and OROS-MPH but showed important limitations (20, 21). The first study was restricted to adolescents (13–17 years old) and excluded those with psychiatric comorbidities (20). The second was a phase 3 study of LDX in children and adolescents with OROS-MPH as a reference arm but cannot be considered a fair head-to-head trial by reason of its design (21). In their network comparative metanalysis, Cortese et al. showed that amphetamines are the first choice of medication in adults compared to methylphenidate (22, 23). Although a pediatric clinical trial analyzing LDX and MPH is currently ongoing (21, 24), more studies comparing these treatments are needed on the pediatric and adult population with ADHD and comorbidities. *In silico* studies have been performed on treatments for ADHD to identify new targets (25) and to investigate their mechanisms of action (MoA) at a structural level (26). However, none has undertaken a quantitative systems pharmacology (QSP) approach to perform a mechanistic comparison between two drugs. Besides, in the mental health field, were investigations on personalized medicine are scarce, recent studies in ADHD have shown how an individualized approach could be beneficial for managing these patients (27, 28).

On the other hand, regulatory agencies have encouraged the usage of computer modeling and simulation approaches to reduce clinical trials time and cost (29). Among these modeling strategies, *in silico* clinical trials (ISCT) provide a platform to test hypotheses on drugs and diseases while reducing risks for patients and the use of animal models (30). We recently published the methodology to develop a mechanistic ISCT using the TPMS technology (with LDX and MPH as a proof-of-concept), which was generated through three phases comprising (i) the molecular characterization of drugs and pathologies, (ii) the generation of a virtual population of 2,600 individuals and the creation of physiologically based pharmacokinetic (PBPK) and QSP models, and (iii) data analysis with artificial intelligence methods (31).

Herein, we analyzed the results of this *in silico* method for comparing modeled or virtual LDX and MPH (hereafter abbreviated as vLDX and vMPH) as ADHD treatments.

The objective was to evaluate the model’s output, considering the models characteristics and the information used to build them, to compare both virtual drugs’ mechanisms and to assess how demographic (age, body mass index, and sex) and clinical (comorbidities) characteristics may affect vLDX’s and vMPH’s relative efficacies, by means of the mathematical models generated.

## 2. Methods

### 2.1. Study design

In this ISCT, we implemented a double-blind crossover-like design simulating a one-year treatment with each drug. The study design, the methodology for building the population and QSP patient-specific models, and the description of the models herein analyzed have been published and extensively described (31). The QSP modeling approach encompassed: the generation of virtual randomized populations following randomized clinical trials’ characteristics (21, 32, 33) and reference population distribution (34, 35), a PBPK modeling approach based on a 14-compartment model considering demographical variation in drug distribution (age, sex, and morphometric measures) (36–39), and a systems biology-based modeling approach using Therapeutic Performance Mapping System (TPMS) technology (40), which mimics the human pathophysiology at a protein-network level using machine learning algorithms to include clinical variation [eight virtual patient profiles considering five common ADHD comorbidities, alone or in combination: namely depression, anxiety, bipolar disorder, tics, and binge eating disorder (BED), and BED + anxiety, BED + depression and depression + anxiety; molecular definition of each comorbidity can be found in Supplementary Table A] and drug mechanisms of action. The method provided virtual patient-specific QSP models of virtual drugs (i.e., defined and modeled according to information from available literature) that complied with the accuracy and quality measurements set for each step (31).

### 2.2. Participants and interventions: defining virtual ADHD patients and virtual drugs

To define the protein network around the area of interest, we performed a bibliographically based molecular characterization of the pathology (ADHD) and the drugs, applying the procedure described in Gutiérrez-Casares et al. (31), to be used as input for TPMS models (40). We defined ADHD as four motives (or biological processes) that describe the pathophysiology of the disease: neurotransmitter imbalance, neuroinflammation, circadian system imbalance, and altered neural viability (31). We only included proteins for which a functional role on the disease was reported. We used expression data to explore ADHD molecular definition variability in Gene Expression Omnibus (GEO) patients, and estimate the minimum population size able to distinguish between healthy and pathological individuals with a 95% statistical power (31); 71 was set as the minimum population size per cohort. Thus, we generated two virtual populations of 1,300 adults and 1,300 children-adolescents with and without ADHD comorbidities (with at least 100 virtual patients per virtual patient profile and 500 virtual patients in the main ADHD population without comorbidities; Supplementary Figures A,B in the Supplementary material S1).

We created randomized virtual populations as previously described (31) using real clinical ADHD trials as references (21, 32, 33). Briefly, population in terms of demography, clinical description, responses to the treatments and clinical trial phase, III in this case, were generated as to mimic demographic and patient characteristics of previous clinical trials with an inclusion/exclusion criterion with a 5% tolerance.

We also used standard European demographic population information (34, 35) to obtain randomized patient distributions for filling missing information from reference trials (Supplementary Tables B,C in the Supplementary material S2 for patient distribution and comparison to reference population).

We used an optimized ADHD definition based on the TPMS tSignal as a proxy of clinical efficacy; this optimization was based on adjusting the definition to QSP mechanisms of different ADHD drugs and to their clinical performance measured with the ADHD-Rating Scale IV (31). Besides, we defined the virtual drugs (vLDX and vMPH) by their protein targets (those proteins for which the drug had activity either *in vitro* or *in vivo*) according to a bibliographical analysis, as described in Gutiérrez-Casares et al. (31) (Table 1) and by the drug’s concentration curve (31) obtained from adjusting a PBPK model (36–39) to reported PK parameters (oral administration; kidney as main clearance organ; bioavailability: 96.4% for Elvanse®, 30% for Medikinet®, and 32% for Concerta®) and real concentration data (51–54). Using this approach, a drug concentration curve per virtual patient was obtained considering their individual characteristics (weight, height, age, and sex).

**Table 1:** Identified protein targets for lisdexamfetamine and methylphenidate (31).

Gene name	Protein name	Effect^*^	Reference of LDX target	Reference of MPH target
TAAR1	Trace amine-associated receptor 1	1	(41)	-
SLC18A2	Synaptic vesicular amine transporter (VMAT2)	-1	(41, 42)	-
SLC6A3	Sodium-dependent dopamine transporter (DAT)	-1	(42–44)	(45–47)
SLC6A2	Sodium-dependent noradrenaline transporter (NET)	-1	(42, 44)	(45–47)
SLC6A4	Sodium-dependent serotonin transporter (SERT)	-1	(42)	-
MAOA	Amine oxidase (flavin-containing) A	-1	(44, 48)	-
MAOB	Amine oxidase (flavin-containing) B	-1	(44, 48)	-
HTR1A	5-hydroxytryptamine receptor 1A	1	-	(49, 50)

* Effect refers to the drug’s action on the protein, 1 denotes activation of protein function, −1 inhibition of protein function.

LDX, lisdexamfetamine; MPH, methylphenidate.

### 2.3. QSP models: outcomes and measures

As previously described (31), through the use of clinical efficacy values for various drugs tested in ADHD clinical trials, modeled drug concentration curves were used to obtain restrictions on target inhibition with ADHD modulation. These restrictions were compatible with systems biology-based TPMS models (40) and conferred them a quantitative dimension. Thus, we obtained a TPMS-derived QSP model per each virtual patient and each virtual drug.

The baseline for modeling was set to the initial status of patients in the recruitment visit, as described in the corresponding real clinical trial. After that, the generated models had the objective of simulating the final patient status after being treated by their corresponding treatments. We included a threshold criterion for accuracy regarding the generated virtual patients’ models. Mathematical solutions with low accuracy (>85% of accuracy) were excluded from the final model.

We retrieved the predicted protein activity (ranging from −1 to 1) from each QSP model (40). We analyzed these data for each protein individually and calculated the tSignal (31, 40), defined as the mean predicted protein activity for the ADHD protein set in each model. We defined a *reverted protein* as one whose activation sign in ADHD was reverted due to the effect of any of the two studied virtual drugs. We considered a protein reverted if the absolute value of its predicted protein activity was higher than 0.5. We defined two further categories of *reverted proteins*: *differentially reverted proteins* were those that presented statistical significant differences in their activity values between the two drugs (see *Statistical analysis* section); *most strongly reverted proteins* were those differentially reverted that were also able to correctly classify and distinguish the patients’ model-related mathematical solutions of the different drugs with 100% accuracy (see *Statistical analysis* section). MoAs (understood as protein paths between the model stimulus and response) explaining the mechanistic involvement of the *most strongly reverted proteins* by each drug were obtained from TPMS models, as previously described (40, 55). We analyzed changes in the tSignal from baseline to compare the efficacy in the comorbid branches. Efficacy is shown as a percentage of the efficacy achieved in the main ADHD population without comorbidities.

### 2.4. QSP models: sensitivity analysis

To analyze the sensitivity of the virtual drugs’ models for each of the protein targets with respect to the model outcome (i.e., ADHD), we carried out a stimulus sensitivity analysis. Similar to a local SOBOL analysis approach (56), we defined the tSignal of the TPMS models as a function of the virtual drugs’ protein targets (model stimulus):

(1) $$tSignal=TPMS\left(X\right)\;for\;X=\left\{X_{1},\;X_{2},\;X_{3},\;\ldots ,\;X_{n}\right\}$$

Where *X* is the model’s stimulus set, or protein target set, and $X_{i}$ each of the protein targets and the modulation (activation/inhibiton) induced by the drug. Then, we evaluated the outcome tSignal variation (expressed as a percentage of the tSignal achieved in the model, where 100% corresponded to the original model and maximal signal intensity associated for each target) for different $X_{i}$ intensities, with variations of one target at a time ranging from 0 to 100% of the effect of the virtual drug over the target using intervals of 25% variation. We evaluated the model output by measuring the tSignal in the optimized-ADHD protein set. This can be expressed as:

(2) $$\frac{d\left(tSignal\right)}{d\left(X_{i}\right)}=\frac{dTPMS\left(X\right)\;}{d\left(X_{i}\right)}$$

Additionally, and because the number of virtual drugs’ protein targets differed, we also evaluated the ADHD-tSignal variation for extended stimulus sets. To that end, the original target protein sets of each virtual drug were extended using random proteins from within the models. A total of 50 different extended stimulus sets were generated at every *X*_*i*+*j*_ protein addition, until reaching 20 additions, *X*_*i*+20_.

These analyses were performed separately for both adult and children-adolescent virtual ADHD populations, using a reduced, random subset of QSP 1,250 TPMS model associated mathematical solutions (5% of the total mathematical solutions for each population).

### 2.5. Statistical analysis

We used descriptive statistics [percentage (%) for categorical variables and mean ± SD for continuous variables] to describe the virtual patient characteristics distribution. We evaluated normality of the data using the Jarque-Bera test, and we used parametric tests to compare normally distributed data and non-parametric tests for non-normal distributions (vLDX vs. vMPH comparison in differentially reverted predicted protein activity, and comorbidity and co-treatment effect). We used Wilcoxon rank sum test to define *differentially reverted proteins*, and a previously described data science strategy (40) to evaluate their classification potential and identify the *most strongly reverted proteins*. In the comparative impact of comorbidities on vLDX and vMPH efficacies over ADHD in adults and pediatric-adolescent population, we used Wilcoxon rank sum test and categorized results according to statistically significance (<0.05 and *p* < 0.001). We evaluated whether the tSignal after co-treatment administration was different from the tSignal with vLDX or vMPH alone (Student’s T test or Wilcoxon rank sum test) and whether this difference was significant when compared to a random drug pool (i.e., the drug database DrugBank). We compared data distributions with Matlab functions and Python or R packages. For protein predicted activity functional evaluation, we used hypergeometric enrichment analysis. For all tests, we adopted the Benjamini-Hochberg false discovery rate (FDR) for multi-test correction and set statistical significance at FDR <0.05, unless otherwise stated. Artificial intelligence methods using a data science strategy (40) were exploited to evaluate predicted protein activity as classifiers.

## 3. Results

### 3.1. Study of the TPMS-based QSP models sensitivity to the drug target sets definition

**Figure 1:**
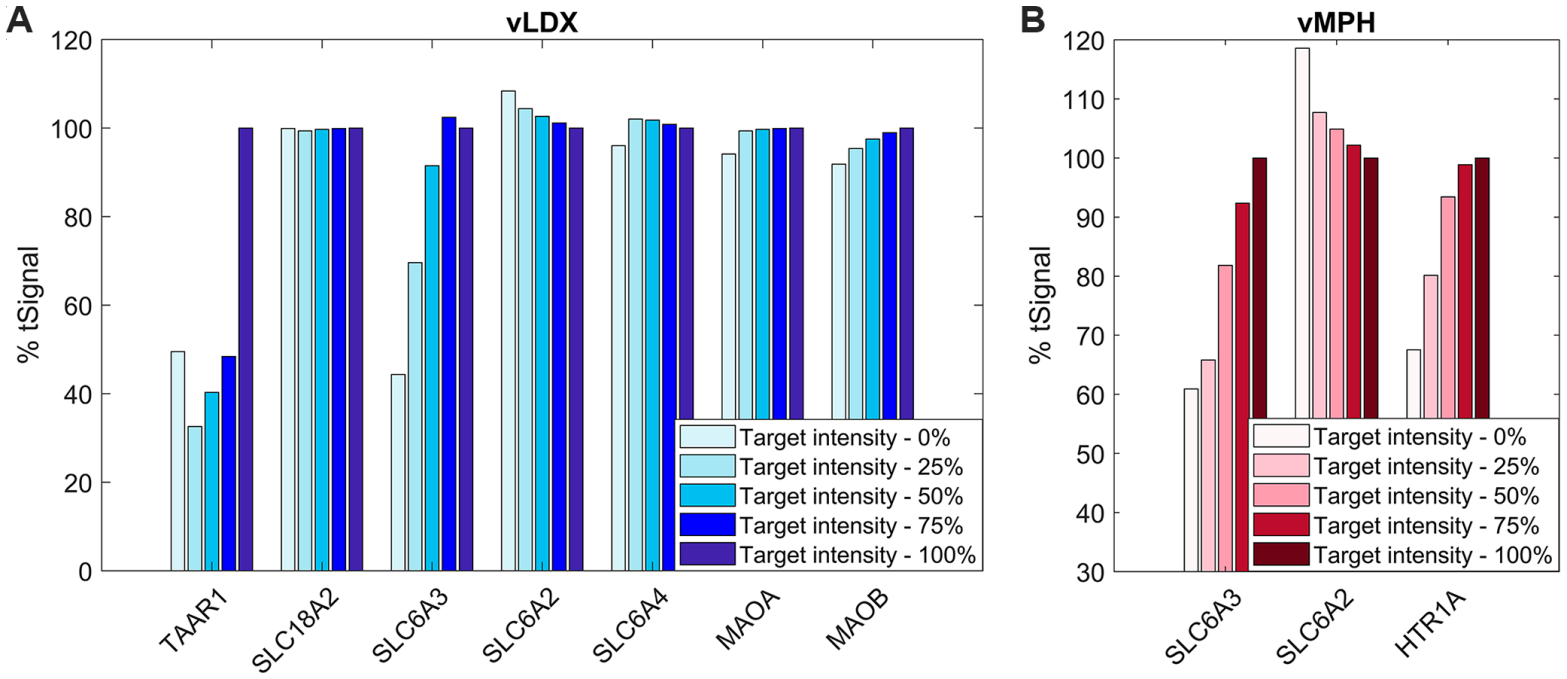
% tSignal with respect to reference adult ADHD TPMS models, that is, the relation between the target-intensity-variated (from 0 to 100%) tSignal calculation with respect to the original model’s mean tSignal. **(A)** vLDX; **(B)** vMPH. Similar results were obtained for the children-adolescent ADHD TPMS model solutions results (data not shown). ADHD, attention-deficit/hyperactivity disorder; TPMS, therapeutic performance mapping system; vLDX, virtual lisdexamfetamine; and vMPH, virtual methylphenidate.

To evaluate the role of the protein targets for both virtual drugs, we applied a sensibility analysis based on a SOBOL strategy, varying the modulation of one target at a time from 0 to 100%. Figure 1 shows the tSignal in the optimized-ADHD protein results, evaluated on adult models’ mathematical solutions and expressed as percentage variation from the tSignal when considering the complete protein target set. In general, a positive intensity-tSignal dependence was observed, leading to lower tSignal than the original models for decreasing intensities.

In the case of vLDX, a strong relation of both TAAR1 and DAT (or SLC6A3) modulation for high ADHD-tSignal variation (reaching >50% variation) was observed, suggesting a relevant role of these targets in the vLDX predicted mechanisms to treat ADHD. For vMPH, intensity variations in both DAT and HTR1A resulted in similar, strong decreased % tSignal with respect to the original models.

To evaluate the model robustness regarding the number of targets included in the virtual drugs’ definitions, we evaluated the influence of using extended protein target sets by adding random proteins as targets to measure the effect on the tSignal of ADHD models. In both cases, a decrease in % of tSignal for larger target sets was detected as new random proteins from the models were added to the stimulus virtual drugs, with a slightly more pronounced effect for vLDX (Supplementary Figure C in the Supplementary material S1).

### 3.2. Virtual drugs’ effects on ADHD pathophysiology

For the sake of conciseness and given the similarity of the results in children-adolescents and adults, figures and tables with our results in children-adolescents are available in Supplementary material S1, S2.

**Figure 2:**
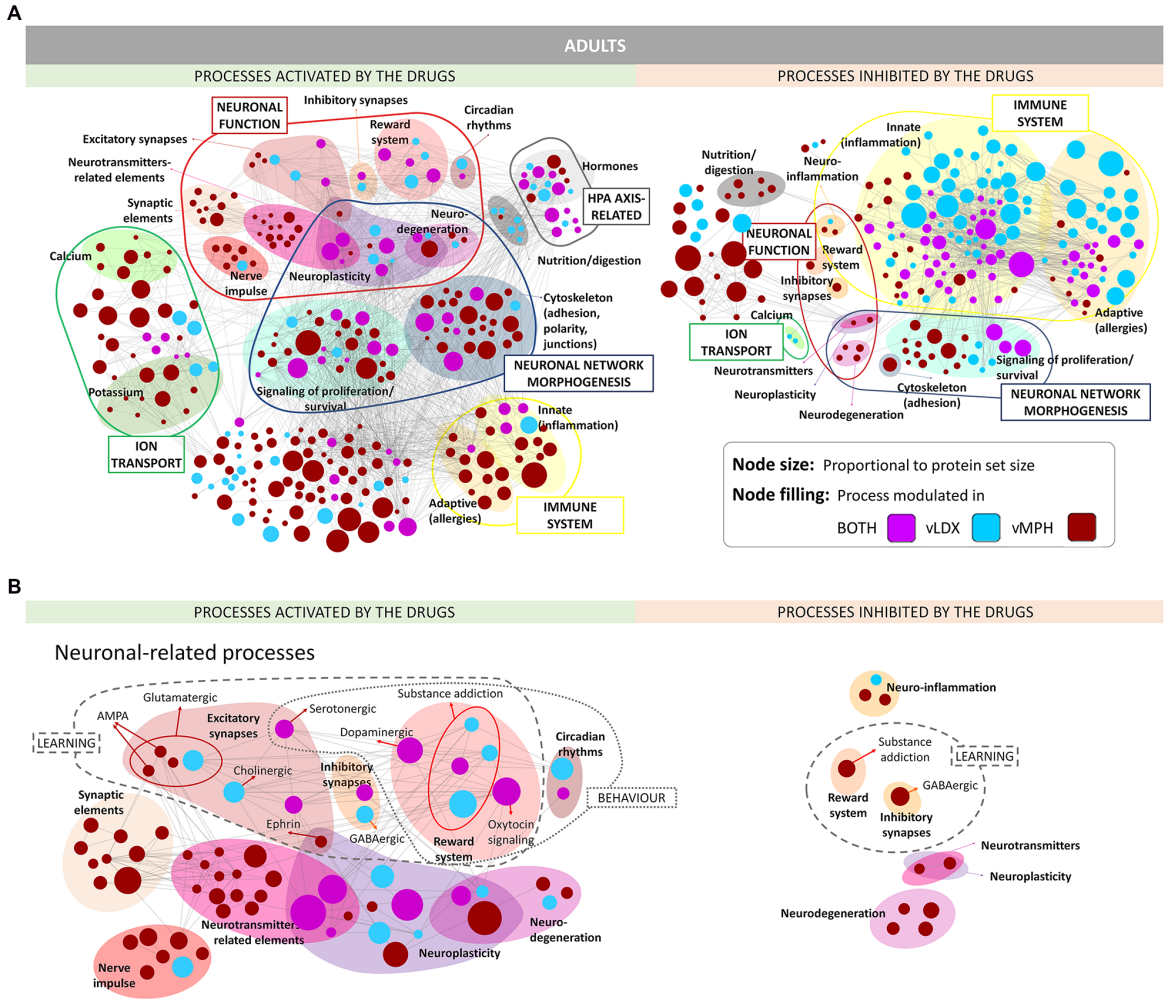
ADHD pathophysiology processes modulated (activated or inhibited) by vLDX and vMPH in adults (see Supplementary Figure D in the Supplementary material S1 for pediatric-adolescent models). **(A)** General overview. **(B)** Neuronal-related processes. ADHD, attention-deficit/hyperactivity disorder; AMPA, γ-amino-3-hydroxy-5-methylisoxazole-4-propionic acid; vLDX, virtual lisdexamfetamine; vMPH, virtual methylphenidate.

Enrichment analyses of the proteins reverted by each drug were performed to functionally profile vLDX’s and vMPH’s MoA models (Figure 2; Supplementary Figure D in the Supplementary material S1). Both drugs modulated ADHD-related defective synapses involved in learning and behavior processes and neuroinflammation and neuroplasticity, including neuronal function and ion transport processers, neuronal network morphogenesis, hypothalamic–pituitary–adrena0A) axis, and the immune system. In addition, vMPH activated several general synaptic, neurotransmitter, and nerve impulse-related processes, suggesting a higher excitatory potential for this drug. On the contrary, vLDX seemed to modulate neural processes more specific to ADHD, including GABAergic inhibitory synapses and regulation of the reward system, as well as a stronger inhibition of inflammatory or immune processes. No relevant differences in these modulated processes were detected between adults and children-adolescents.

### 3.3. Mechanisms of action of virtual drugs

**Figure 3:**
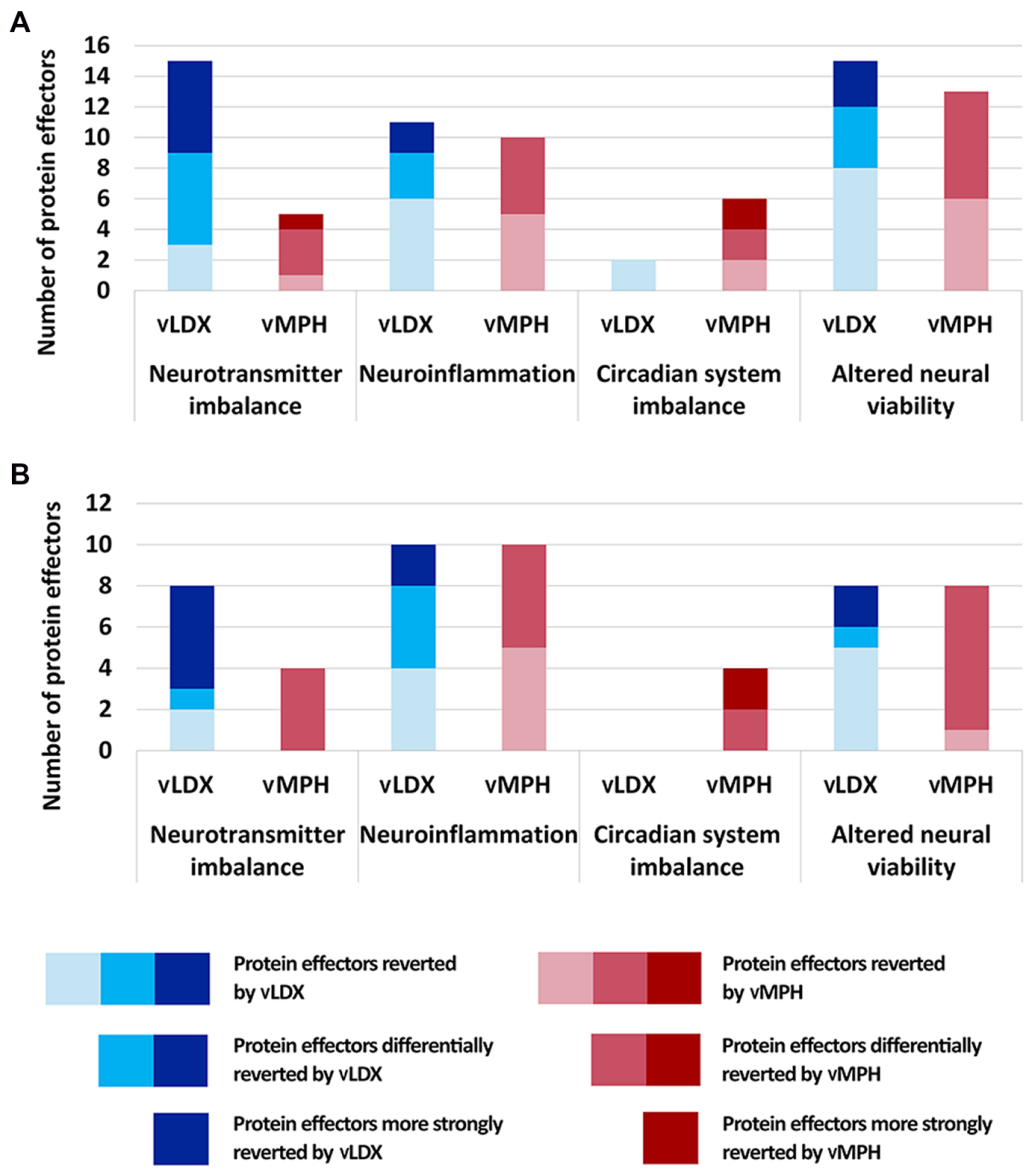
Number of protein effectors reverted by vLDX and vMPH classified according to the four pathophysiological processes involved in ADHD **(A)** in adults and **(B)** in children. ADHD, attention-deficit/hyperactivity disorder; vLDX, virtual lisdexamfetamine; vMPH, virtual methylphenidate.

Virtual LDX strongly modified neurotransmitter imbalance, neuroinflammation, and altered neural viability, whereas vMPH had a significant impact on circadian system imbalance, as well as on neuroinflammation and altered neural viability (Figure 3). In adults, the vMPH effect on neuroinflammation and altered neural viability-related processes was more marked than in children and adolescents. However, the efficacy of vLDX in both populations was almost identical, although the most modulated motive in adults was neuroinflammation and the one in children-adolescents was neurotransmitter imbalance. Generally, models of ADHD treatment showed a stronger tSignal when treated with vLDX than with vMPH (0.25 ± 0.01 vs. 0.13 ± 0.01 in adults, 0.31 ± 0.04 vs. 0.08 ± 0.01 in children-adolescents, FDR < 0.01). However, both drugs modulated to a certain degree the four ADHD pathophysiological processes (Figure 3), except for vLDX over circadian system imbalance in children-adolescents.

**Figure 4:**
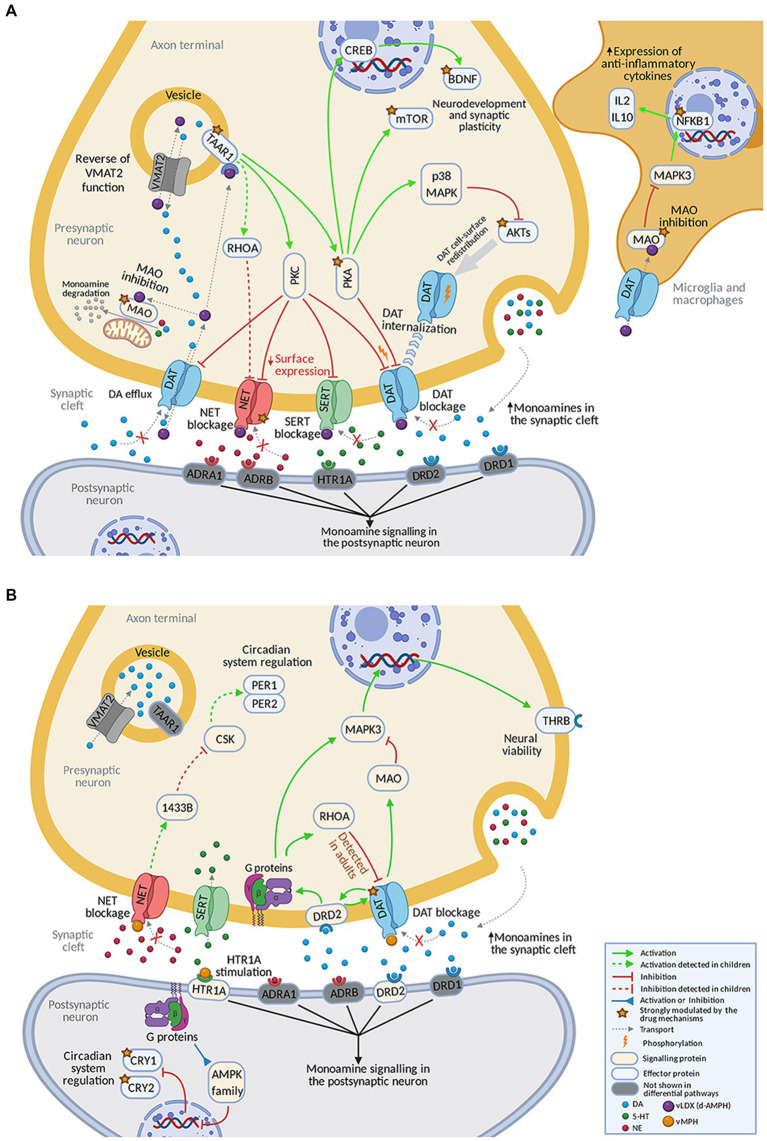
Predicted mechanism of action of **(A)** vLDX and **(B)** vMPH in ADHD. Supplementary Figure E in the Supplementary material S1 and Supplementary Tables D,E in the Supplementary material S2 contain the sources of information found in the scientific literature supporting the predicted mechanisms. Created with BioRender.com. ADHD, attention-deficit/hyperactivity disorder; vLDX, virtual lisdexamfetamine; vMPH, virtual methylphenidate.

The mechanisms behind the strongly reverted effectors by each drug were explored (Figure 4). vLDX differential mechanisms were found to be mediated by the inhibition of the activity of five proteins involved in the monoaminergic system: dopamine (DAT1), noradrenaline (NET1), and serotonin (SERT) transporters, monoamine oxidase (MAO) and, mainly, trace amine-associated receptor 1 (TAAR1; Figure 4A). In our models, vLDX agonistic effect over TAAR1 activated downstream PKC/PKA. vLDX strongly modulated NET1 and FLOT1 by downregulating NET1 and SERT through TAAR1-mediated PKC activation. The latter could also induce p38 MAPK-mediated inactivation of AKTs. Also, TAAR1-induced PKA stimulation was predicted to cause the expression of BDNF through CREB activation. Furthermore, MAO inhibition in inflammatory cells could signal through MAPK3 and NF-KB (NFKB1), in turn inducing the expression of anti-inflammatory cytokines (e.g., IL2 and IL10).

According to our model, the differential effects of vMPH were mediated by the inhibition of DAT1, agonism of the 5-HT1A receptor, and subsequent inhibition of circadian clock regulators CRY1 and CRY2 (Figure 4B). vMPH affected kinases such as RhoA, the AMPK family, and MAPK3 (ERK1) through HTR1A receptor agonism and indirect modulation of dopamine 2 (DRD2) receptor by dopamine accumulation. On the other hand, DAT1 inhibition led to a reduction in MAO activity. vMPH also stimulated the thyroid receptor (THRB) expression through MAPK3. Moreover, a presynaptic effect over circadian proteins PER1 and PER2 through NET blockade was detected in children-adolescents. In children, a differential inhibition of NET was observed for vLDX, and a small difference, the reinforced inhibition of DAT1 by RhoA, was detected for vMPH.

### 3.4. Effects of demographic parameters

**Figure 5:**
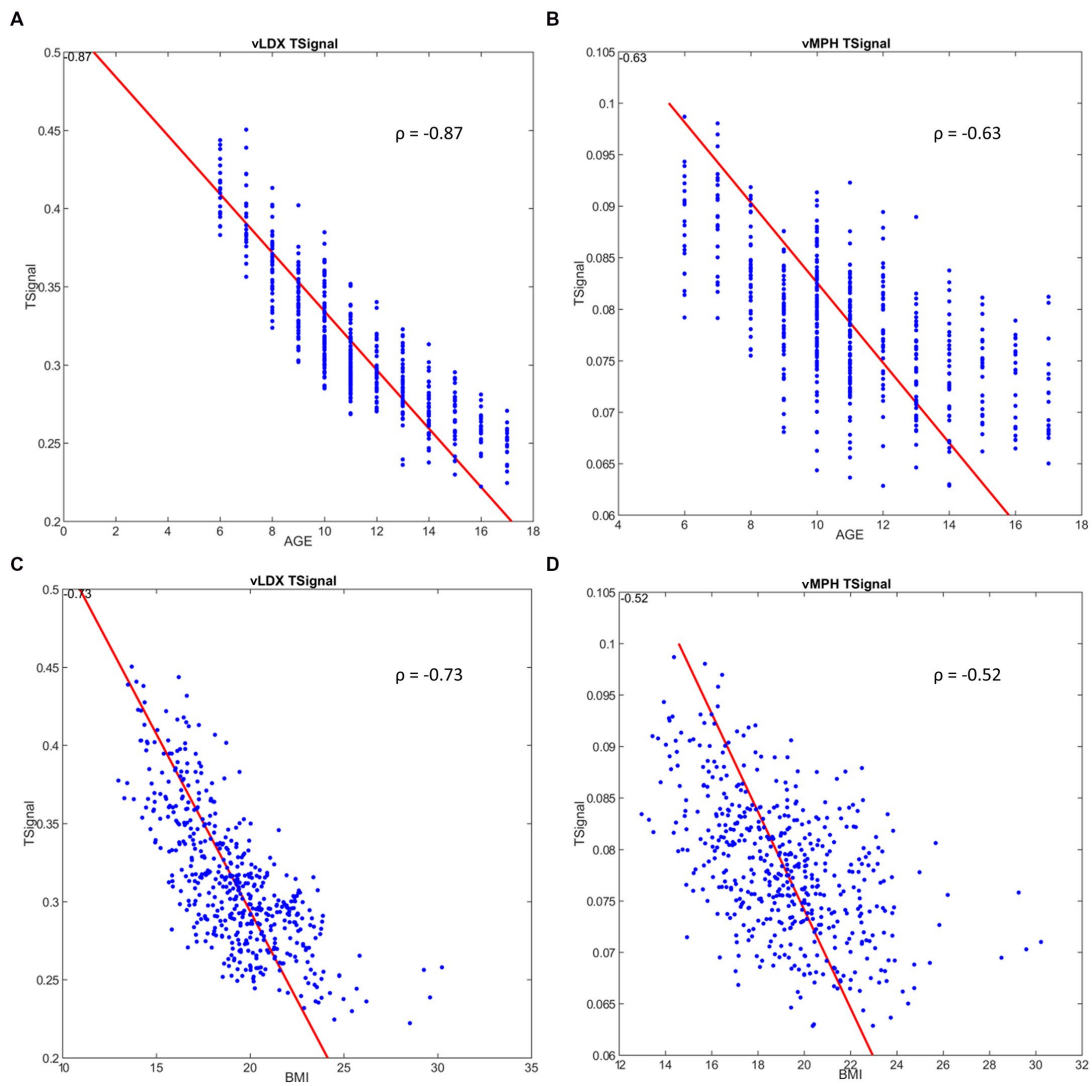
Correlation between ADHD tSignal and age or BMI in the pediatric-adolescent population. **(A)** ADHD tSignal in vLDX mechanistic models vs. age; **(B)** ADHD tSignal in vMPH mechanistic models vs. age; **(C)** ADHD tSignal in vLDX vs. BMI; **(D)** ADHD tSignal in vMPH vs. BMI. ADHD, attention-deficit/hyperactivity disorder; BMI, body mass index; vLDX, virtual lisdexamfetamine; vMPH, virtual methylphenidate; ρ, Pearson’s correlation coefficient.

According to our models, age did not affect vLDX’s nor vMPH’s efficacy in adults (Table 2). However, younger age was associated with better efficacy for both treatments in the pediatric-adolescent population, albeit more significantly with vLDX (Table 2; Figure 5). Body mass index (BMI) seemed to impact negatively on vLDX’s efficacy in all the populations (Table 2), but especially in children-adolescents (Figure 5).

**Table 2:** Summary of the impact of vLDX and vMPH on demographic characteristics of adults and children-adolescents with ADHD.

Demographic characteristics	Children (6–12 years)		Adolescents (13–17 years)		Adults	
	vLDX	vMPH	vLDX	vMPH	vLDX	vMPH
Age^a^ (ρ)	Strong (−0.85)	Moderate (−0.53)	Moderate (−0.58)	–	–	–
BMI^a^ (ρ)	Moderate (−0.53)	Weak (−0.49)	Moderate (−0.67)	–	Weak (−0.33)	–
Sex^b^	–	Male	Male	–	Female	Female

a Correlation strength (ρ) calculated using Pearson’s correlation method. False Discovery Rate (FDR) computed as Benjamini-Hochberg corrections for multiple testing. Statistically significant correlations were considered when FDR < 0.05 [if not significant, (−) is indicated]. Direction: positive correlation (ρ > 0) meant the higher the parameter result, the higher the efficacy; negative correlation (ρ < 0) meant the higher the parameter, the lower the efficacy. Strength: Strong: |ρ| ≥ 0.8; Moderate: 0.8 > |ρ| ≥ 0.5; Low: 0.5 > |ρ| ≥ 0.3; Negligible: |ρ| < 0.3 [not shown (−)].

b Cohort displaying the highest efficacy. Calculated through unpaired two-tailed Student’s T test or Wilcoxon rank sum test, depending on the distribution in each cohort. FDR computed as Benjamini-Hochberg corrections for multiple testing. Only statistically significant results are shown [FDR < 0.05; if not significant, (−) is indicated].

ADHD, attention-deficit/hyperactivity disorder; BMI, body mass index; vLDX, virtual lisdexamfetamine; and vMPH, virtual methylphenidate.

A weak impact of BMI was detected for vMPH in the pediatric-adolescent population (Figure 5), mainly in children (6–12 years; Table 2). This result could be associated with the effects of age on efficacy, especially in children with low BMI, since after isolating age from BMI variability, age continued to correlate moderately to vMPH’s efficacy when BMIs were low, whereas BMI did not maintain its moderate correlation when isolating by age (Supplementary Figure F in the Supplementary material S1). On the other hand, the impact of BMI and age on vLDX’s efficacy seemed to be independent, as a moderate and strong correlation was detected when isolating by either age or BMI (Supplementary Figure G in the Supplementary material S1). Furthermore, the efficacies of vLDX and vMPH were predicted to be greater on adult females than on males, whereas, on children-adolescents, males seemed to be better respondents to both treatments.

### 3.5. Effects of comorbidities

**Figure 6:**
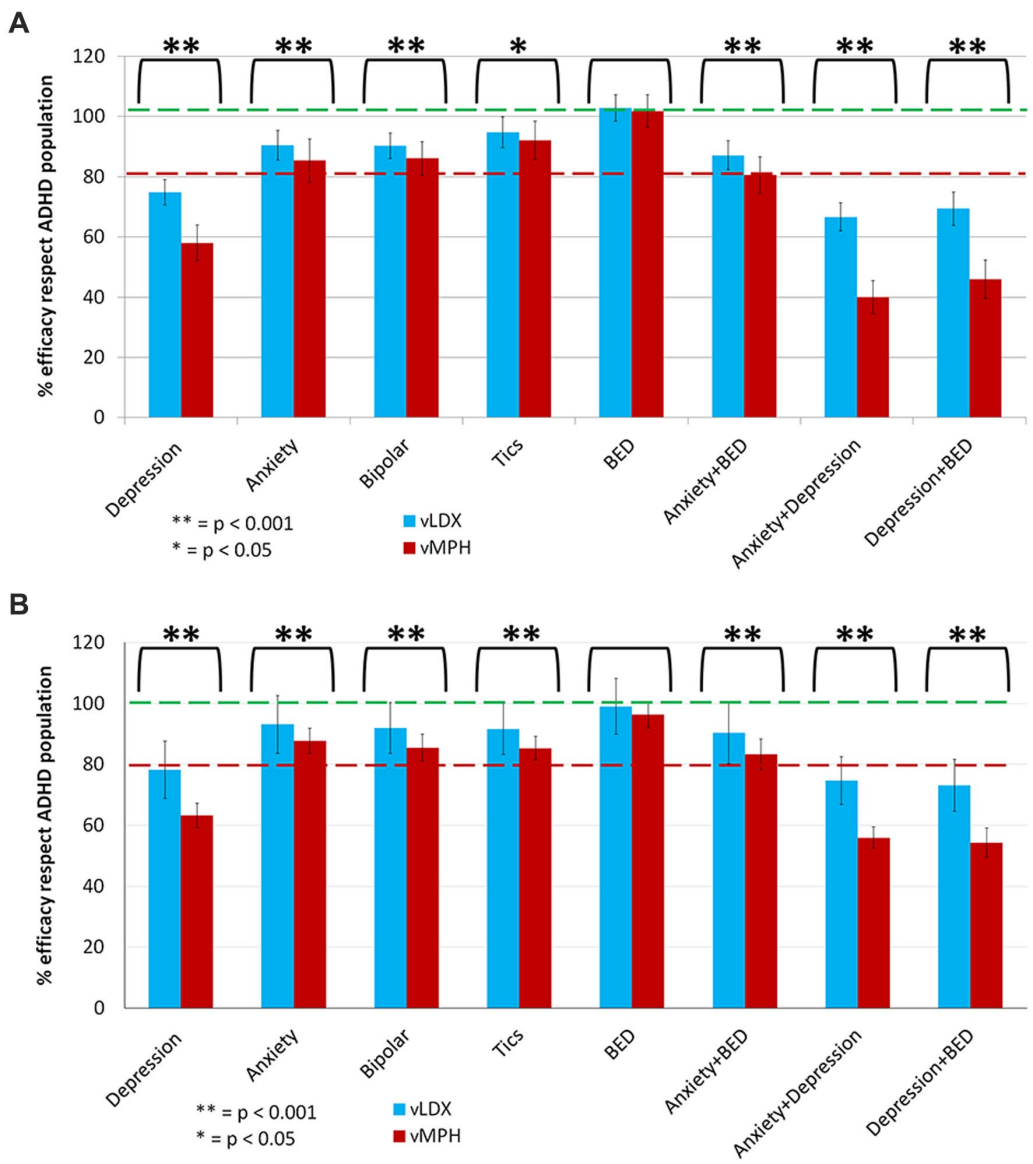
Summary of the comparative impact of comorbidities on vLDX and vMPH efficacies over ADHD in **(A)** adults and **(B)** pediatric-adolescent population. ADHD, attention-deficit/hyperactivity disorder; BED, binge eating disorder; vLDX, virtual lisdexamfetamine; vMPH, virtual methylphenidate.

In our models, depression negatively impacted both drugs’ efficacies, although to a lesser degree on vLDX (Figure 6). BED did not affect either the efficacy of vLDX or vMPH, as neither did the remaining studied comorbidities (i.e., anxiety, bipolar disorder, and tic disorders).

### 3.6. Effects of co-treatments

In adults, our results showed that the co-treatments that affected vLDX’s efficacy were those associated with tic disorders management (guanfacine, aripiprazole, tiapride, haloperidol, and risperidone). In contrast, vMPH’s efficacy seemed to be susceptible to wide-spectrum psychiatric drugs used for the treatment of the five studied comorbidities (Table 3). Although the impact on efficacy was significant (significant difference in the tSignal), this difference was not significant compared to a random drug pool. The results were similar for the pediatric-adolescent population (Table 4).

**Table 3:** Summary of the comparative impact on vLDX’s and vMPH’s efficacy of drugs commonly used to treat ADHD comorbidities in adults.

Condition	Drug	vLDX		vMPH	
		Impact^a^	DB reference^b^	Impact^a^	DB reference^b^
Tics	Aripiprazole	<0.01	<0.01	-	-
	Guanfacine	<0.01	<0.05	<0.01	-
	Haloperidol	<0.01	<0.05	-	-
	Risperidone	<0.01	<0.05	-	-
	Tiapride	<0.01	<0.01	-	-
AnxietyBip. disorderDepression	Citalopram/Escitalopram/Fluvoxamine/Paroxetine	-	-	<0.01	-
	Sertraline	-	-	<0.01	-
Bip. disorder	Lithium	<0.01	-	<0.01	-
DepressionBinge eatingBip. disorder	Fluoxetine	<0.01	-	<0.01	-

a Calculated by comparing the tSignal when treating only with the drug of interest or with the drug + co-treatment. Calculated through unpaired two-tailed Student’s T test or Wilcoxon rank sum test, depending on the distribution in each cohort.

b Calculated against the distribution of drugs stored in DrugBank.

Bip. Disorder, bipolar disorder; DB, DrugBank; vLDX, virtual lisdexamfetamine; and vMPH, virtual methylphenidate.

**Table 4:** Summary of the comparative impact on vLDX’s and vMPH’s efficacy of drugs commonly used to treat ADHD comorbidities in children-adolescents.

Condition	Drug	vLDX		vMPH	
		Impact^a^	DB reference^b^	Impact^a^	DB reference^b^
Tics	Aripiprazole	<0.01	<0.01	-	-
	Guanfacine	<0.01	<0.01	<0.01	-
	Haloperidol	<0.01	<0.01	-	-
	Risperidone	<0.01	<0.01	-	-
	Tiapride	<0.01	<0.01	-	-
Anxiety	Hydroxyzine	<0.01	<0.01	-	-
Anxiety					
Depression Binge eating Bip. disorder	Fluoxetine	<0.01	-	<0.01	-
Bip. disorder	Lithium	<0.01	<0.01	<0.01	-
Bip. disorder Depression	Citalopram/Escitalopram/Fluvoxamine/Paroxetine	-	-	<0.01	-
	Sertraline	-	-	<0.01	-

a Calculated by comparing the tSignal when treating only with the drug of interest or with the drug + co-treatment. Calculated through unpaired two-tailed Student’s T test or Wilcoxon rank sum test, depending on distribution in each cohort.

b Calculated against the distribution of drugs stored in DrugBank.

Bip. disorder, bipolar disorder; DB, DrugBank; vLDX, virtual lisdexamfetamine; and vMPH, virtual methylphenidate.

## 4. Discussion

In this ISCT between vLDX and vMPH, our models showed that these drugs might present differences in their MoA that could influence their efficacies, particularly in patient groups determined by demographic and clinical characteristics.

To evaluate the sensitivity of the models to the protein target set definition, our stimulus sensitivity analysis showed that, for both drugs, two targets had the most impact over ADHD-signal response: TAAR1 and DAT in vLDX and DAT and HTR1A in vMPH virtual patients. Notably, the slightly higher tSignals observed at lower inhibition modulation intensities, in the case of NET, could be due to a possible additive effect with other targets that might affect tSignal definition. When adding random proteins to the stimulus set, outcome response decreased in both cases, pointing toward a target set characterization importance rather than protein target set size relevance. Therefore, these results indicated that our mathematical models were correctly adjusted according to our initial premises.

As reported in previous studies, we found that vLDX and vMPH acted upon neuronal functions and synapses (18, 19, 41, 57–73). Also, previous reports support our findings on the effect of LDX and MPH on the neuroplasticity/neurodegeneration equilibrium (74–78). Besides, in our models, vLDX inhibited more strongly neuroinflammation than vMPH, in agreement with previous studies but in contrast with others (70, 79–86).

Lisdexamfetamine has been described to target critical proteins involved in the monoaminergic system: DAT1, NET1, SERT, MAO, and the less characterized TAAR1 (87–99), which could mediate its activity. DAT is the main regulator of dopaminergic tone in the central nervous system and is present in lymphocytes and monocytes/macrophages (100). Several studies have found that high-affinity monoamine neurotransmitter transporters, such as DAT, SERT, and NET, are expressed on neuroglia cells (101). Contrary to MPH, LDX has been reported as an agonist of TAAR1, an amine receptor known to modulate the dopamine pathway, though its role in ADHD remains unclear (102, 103). According to our results, this particular feature could give vLDX significant benefits toward ADHD response, while leading to mechanistic differences with vMPH because of the role played by TAAR1 in the brain. Its activation results in a decreased cell surface expression of DAT1, NET1, and SERT (94–96). In addition, amphetamine binding to TAAR1 could reduce the firing rate of the dopamine neuron via potassium channels and activate PKA and PKC, which could subsequently phosphorylate DAT (104, 105). PKA-phosphorylation may cause DAT internalization into the presynaptic neuron and cease transport (104), and PKC phosphorylation could induce the same effect on DAT or the opposite (104). Importantly, amphetamine-stimulated dopamine efflux through DAT has been shown to require PKC activation (106). Through TAAR1-mediated PKC activation, LDX could be modulating more strongly NET1 and FLOT1 than MPH (88, 95, 96, 107). Also, LDX action on TAAR1 might induce inactivation of AKTs and modulation of the AKT/mTOR pathway with direct implications for ADHD (97, 98, 108–111). The TAAR1-mediated stimulation of PKA leads to the expression of BDNF, a neurotrophin with an important role in ADHD (112, 113). The cAMP signaling pathway has been reported to activate CREB through PKA, p38, and MSK1 in NIH 3 T3 cells (112). Once amphetamine has penetrated the neuronal membrane, it could bind to TAAR1 or enter synaptic vesicles through the VMAT2 transporter (104, 114). The latter could cause the collapse of the vesicular pH gradient and dopamine release into the cytosol (114, 115). Our models showed MAO reduced activity due to DAT inhibition, which could be stronger in vLDX due to the TAAR1 modulation effect on DAT, besides the direct effect of vLDX. MAO inhibition could activate MAPK3, involved in neuroinflammation through expression-control of anti-inflammatory cytokines (IL-2, IL − 10) (99). NFKB1 activation has been reported to downregulate MAPK3 in ADHD patients and be more strongly reverted by LDX than by MPH (89, 99, 116). Thus, vLDX-derived activation of TAAR1 reinforced other monoamine-related vLDX effects mediated by targets, improving neurotransmitter regulation and affecting other relevant processes in ADHD (i.e., neuroplasticity, neuronal survival, and neuroinflammation). Additionally, in children-adolescents, the inhibition of the NET1 pathway through TAAR1 via RhoA modulation could explain the differential inhibition of NET in favor of vLDX (90, 117, 118).

The differential mechanism of vMPH strongly inhibited DAT1 and circadian clock regulators CRY1 and CRY2 (50, 119–126). These mechanisms were modeled to occur, among others, by inhibiting DAT1 (119) and AMPK signaling following HTR1A agonism (50). DAT1, DRD1, and DRD2 have been reported to regulate dopamine and physically interact, reciprocally modulating each other’s functions presynaptically (120, 127). However, evidence on DRD2 modulation by MPH is extensive and different conclusions are reached depending on the studied brain regions, possibly because of differences in postsynaptic dopamine concentration and DRD distribution and regulation (128). The activity of MPH has been postulated to increase the concentration of extracellular dopamine via multiple mechanisms, including DAT blockade, disinhibition of DRD2 autoreceptors on the presynaptic dopaminergic neuron (DRD2 autoreceptors remain inhibited with no treatment), and activation of DRD1 receptors on the postsynaptic neuron. This results in an amplification of dopamine activity and improvement of attentional deficits, cognitive functioning, and motor hyperactivity (129). The activation of presynaptic DRD2 after MPH administration may induce redistribution of membrane vesicles resulting in increased dopamine release (130). However, this activation has also been suggested to decrease dopamine release through feedback inhibition (128). Besides, inhibiting DAT1 could lead to a reduced activity of MAO (131, 132) caused by a decrease in intracellular dopamine levels and further activity of MAPK3. Through the stimulation of THRB (123, 133), MPH might improve the altered neural viability in ADHD. In addition, the MPH-mediated activation of HTR1A and DRD2 (and potentially other G-coupled receptors) has been shown to potentially have a reinforcing feedback role on the MPH modulation of neurotransmitter imbalance through further regulation of DAT1 (119). Other than being directly inhibited by MPH, this transporter could also be regulated by the activation of the small GTPase RhoA, which allows the internalization of the DAT1 reuptaker (124). According to our models, the most differentially modulated mechanism by vMPH was the circadian system. CRY1 and CRY2 have been implicated in the ADHD-associated dysregulation of the circadian system (125) and were modeled to be better stabilized by vMPH than vLDX through modulation of AMPK activity (126). In children, the mechanisms modeled for vMPH were almost identical to those of adults.

According to our results, demographic characteristics impacted vLDX’s and vMPH’s efficacy differently in adult and pediatric-adolescent populations. In adults, the impact of demographic characteristics on ADHD treatments has been poorly studied. Our results suggested that only sex, and to a lower extent BMI, seemed to have an impact on the efficacies of vLDX and vMPH in adults. In children, our models showed that age and BMI could affect both virtual drugs’ efficacies, a finding supported by previous reports (134, 135). This correlation was more notable in vLDX, showing a stronger efficacy in the pediatric population (≤12 years old) than in adolescents. Given the importance of the BMI-for-age correlation in the pediatric-adolescent population (35), we evaluated whether the efficacies of vLDX and vMPH varied with age and BMI. While changes in vMPH’s efficacy related to BMI seemed to be associated with an age effect, vLDX’s efficacy was predicted to be affected independently by age and BMI.

Regarding ADHD psychiatric comorbidities, data from the United States population shows that depression is more common in adults (8.1%) than in children-adolescents (3.2%), whereas tic disorders are more prevalent among the latter (136–138). On the other hand, the prevalence of anxiety is similar among children-adolescents (7.1%) and adults (6.7%) (138, 139). Although depression affected both drugs’ efficacies, the effect was smaller on vLDX than on vMPH. No other important effect over the efficacy of ADHD for either drug was detected for any of the remaining analyzed comorbidities. In addition, we found that the efficacy of vMPH could be altered by a class of drugs (selective serotonin reuptake inhibitors) used for a broad range of psychiatric disorders, including four out of the five studied comorbidities (i.e., anxiety, depression, bipolar disorder, and BED). In contrast, only drugs for tic disorders could affect vLDX’s efficacy.

Our models were built on the widest available information (at the molecular and clinical level) around patients, disease, and treatments and our approach provided reference standards to validate each modeling step (31). However, our study was subjected to the intrinsic boundaries of ISCT, bearing both limitations and strengths, and its results must be interpreted considering them. First, the models described here were built upon the current knowledge of human physiology, particularly on the diseases’ and drugs’ definitions, as discussed in previous works (31, 40, 55). This implies that our results could be affected by missing data, errors, and bias, and some aspects could have been overlooked. For instance, to define virtual drugs, we applied equivalent search and selection criteria for both drugs (31), but we did not control for possible literature bias that might affect the information selection and could impact the conclusions. Second, stemming from the previously mentioned constraint, our models were limited by the inherent restraints of mathematical models, which cannot fit 100% of the training data information. However, this approach allows obtaining a diversity of biologically plausible models mimicking the diversity observed in human physiological responses, which could also be considered a strong point of the technique (55). This modeled “molecular diversity,” together with the patient-specific information included through demographic and clinical information, renders this strategy suitable to investigate MoA in a diverse group of virtual patients. This, in turn, enables the raising of hypotheses toward treatment selection through patient segmentation and personalized medicine. Third, our modeling methodology considered only the impact of patient-specific demographic characteristics on the drugs’ absorption, distribution, and excretion. However, other consequences of these characteristics at the MoA level were not considered, such as the role of sex-dependent hormonal differences or age-related neurodevelopment and their effects on the drug’s outcome. Fourth and last, we used reported information on the clinical effect of the studied drugs, but a vast dispersion of values was observed, forcing us to average these values for the training process (31). Notably, although this was a theoretical study with all the implied restraints of such approaches, it could set the ground for generating hypotheses and new research to deepen our understanding of the mechanisms and differences among these drugs. Nonetheless, pre-clinical and clinical validations of the results herein exposed are necessary to extend their applicability to clinical practice. All the limitations described above applied to both vLDX and vMPH models.

## 5. Conclusion

Our ISCT, using MoA models of vLDX and vMPH over virtual ADHD patients, suggested that LDX and MPH have similar efficacy mechanisms and modulate common ADHD pathophysiology processes, but could target different disease mechanisms. The models generated showed that demographic characteristics could have an effect on these drugs’ efficacies, mainly BMI and age in the pediatric population. In addition, comorbidities and their treatment could differentially affect the mechanisms of both drugs to treat ADHD. Although requiring clinical validation, our *in silico* results raised hypotheses that could be strategic for conditioning the experimental design of future clinical or pre-clinical studies and pave the way for personalized medicine and drug selection by patient profiling.

## Data availability statement

The original contributions presented in the study are included in the article/Supplementary material, further inquiries can be directed to the corresponding authors.

## Author contributions

JG-C, JQ, CM, and MC conceived the study. JG-C and JQ performed the investigation. CS-V and MC performed the formal analyses. PR and TP-R managed the project. JG-C and JQ supervised and CM validated the study. PR, TP-R, and CS-V aided in visualization tasks. JG-C, JQ, and CS-V drafted the first version of the manuscript. All authors contributed to the article and approved the submitted version.

## Funding

Takeda funded this study and the medical writing support.

## Conflict of interest

PR was a full-time employee at Takeda at the time of the study. CM and TP-R are full-time employees at Takeda. CS-V and MC are full-time employees at Anaxomics Biotech. JG-C has served as speaker for Takeda and Shire and has received research funding from Shire. JQ has served as speaker and/or on scientific advisory boards for Takeda, Janssen, and Rubio.

The authors declare that this study received funding from Takeda. The funder had the following involvement with the study: study design, contribution to data interpretation, and critical revision of the manuscript.

## Publisher’s note

All claims expressed in this article are solely those of the authors and do not necessarily represent those of their affiliated organizations, or those of the publisher, the editors and the reviewers. Any product that may be evaluated in this article, or claim that may be made by its manufacturer, is not guaranteed or endorsed by the publisher.

## Supplementary material

The Supplementary material for this article can be found online at:

https://www.frontiersin.org/articles/10.3389/fpsyt.2023.939650/full#supplementary-material
