# Supplementary material for: In silico clinical trial evaluating lisdexamfetamine’s and methylphenidate’s mechanism of action computational models in an attention-deficit/hyperactivity disorder virtual patients’ population
Source: Front Psychiatry. 2023 Jun 2;14:939650. doi: 10.3389/fpsyt.2023.939650 (PMC10273406; doi:10.3389/fpsyt.2023.939650)
Supplement: Supplementary file 2 [file Data_Sheet_1.docx]

***Supplementary Figures***

## Supplementary Figures

| 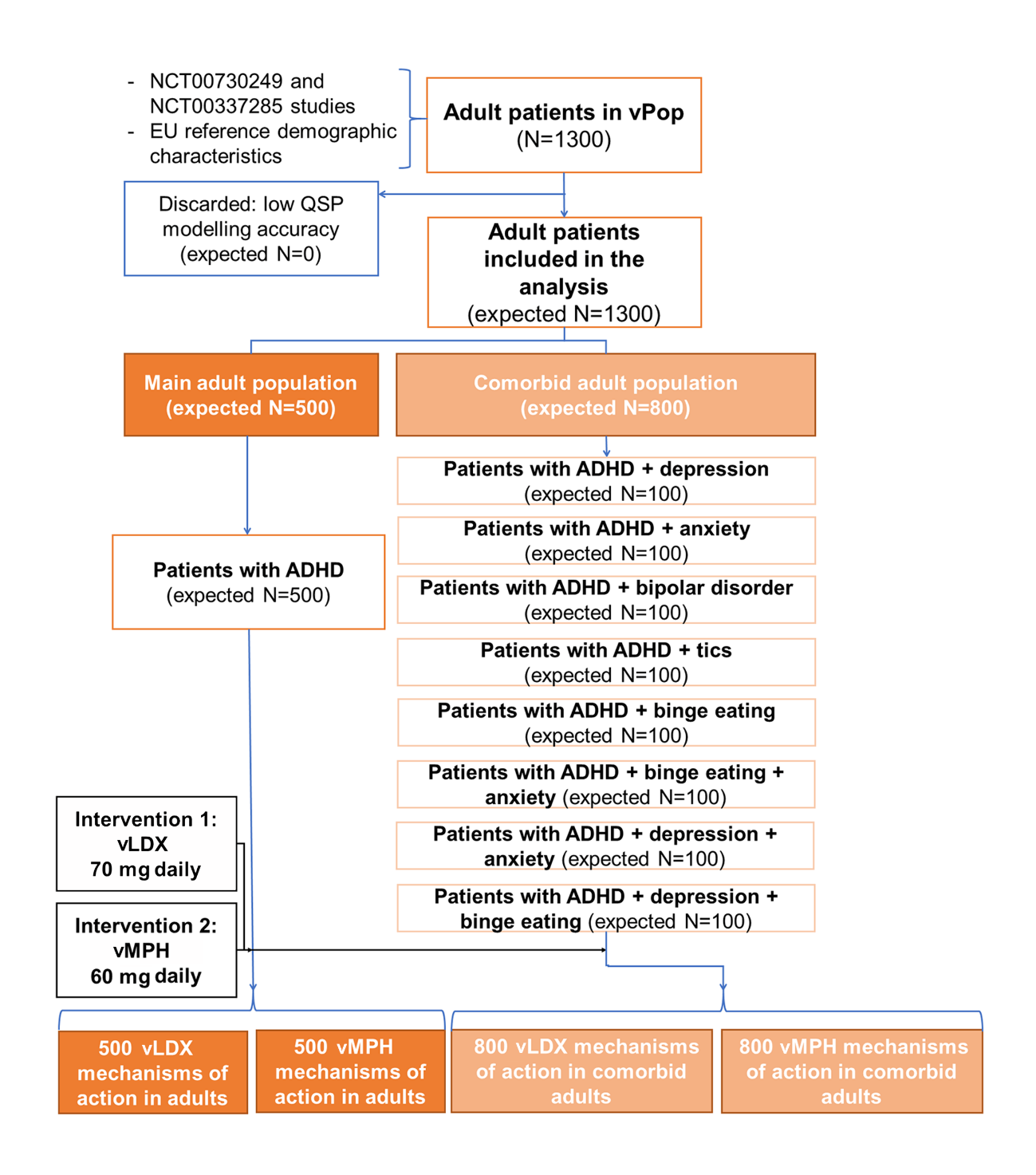 |
| --- |
| **Figure A.** **Comorbidities distribution and treatment allocation in the adult virtual population**  ADHD: Attention-deficit/hyperactivity disorder; QSP: Quantitative systems pharmacology; vLDX: Virtual lisdexamfetamine; vMPH: Virtual methylphenidate; vPop: Virtual population. |
| 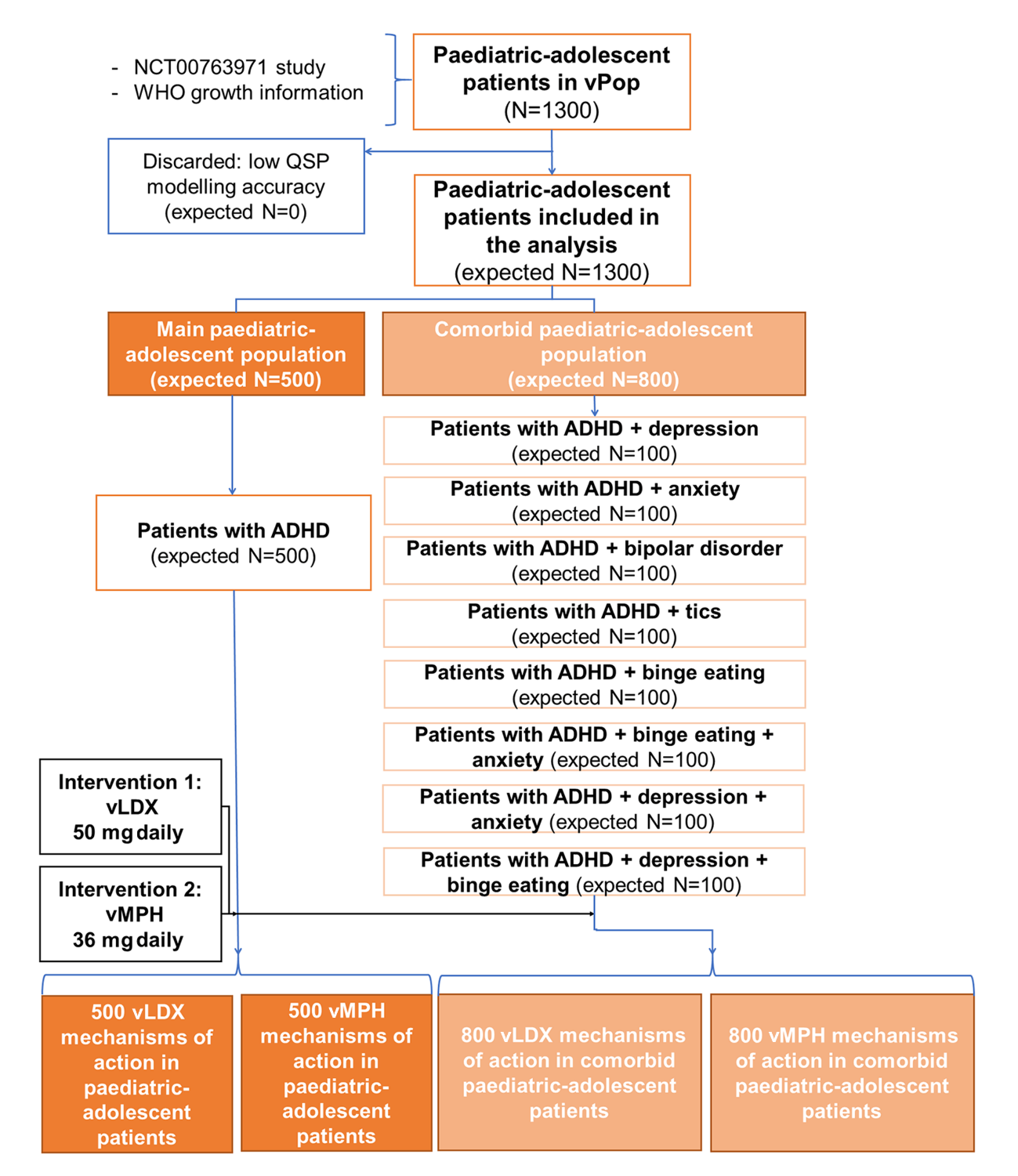 |
| **Figure B.** **Comorbidities distribution and treatment allocation in the pediatric-adolescent virtual population**  ADHD: Attention-deficit/hyperactivity disorder; QSP: Quantitative systems pharmacology; vLDX: Virtual lisdexamfetamine; vMPH: Virtual methylphenidate; vPop: Virtual population. |


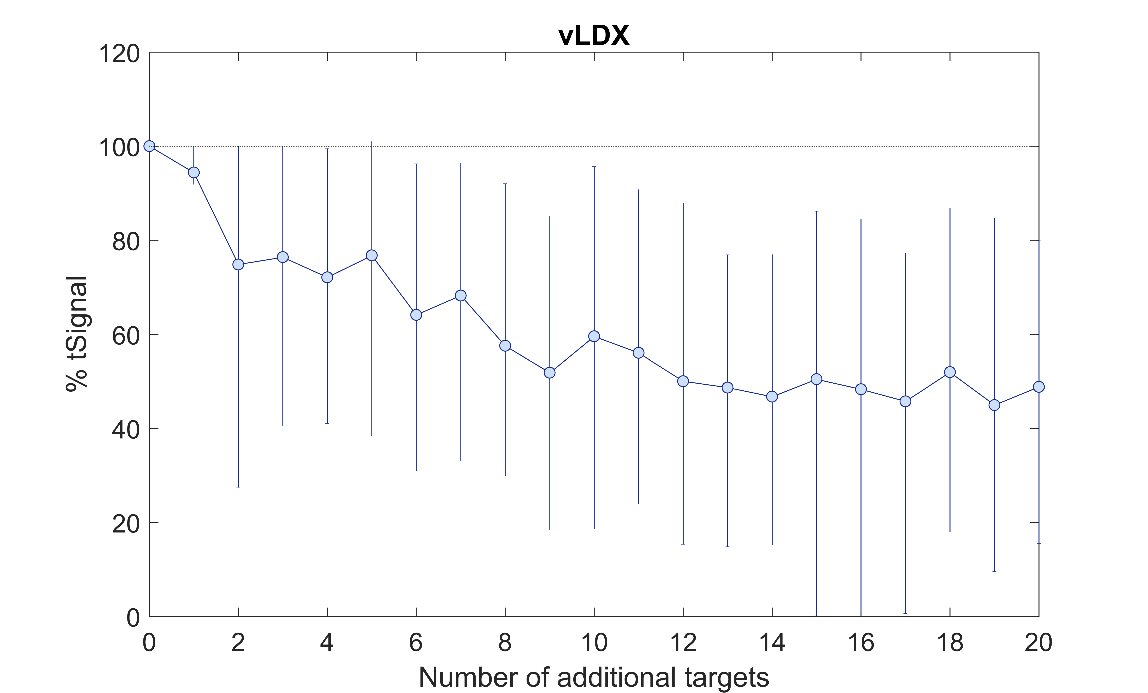

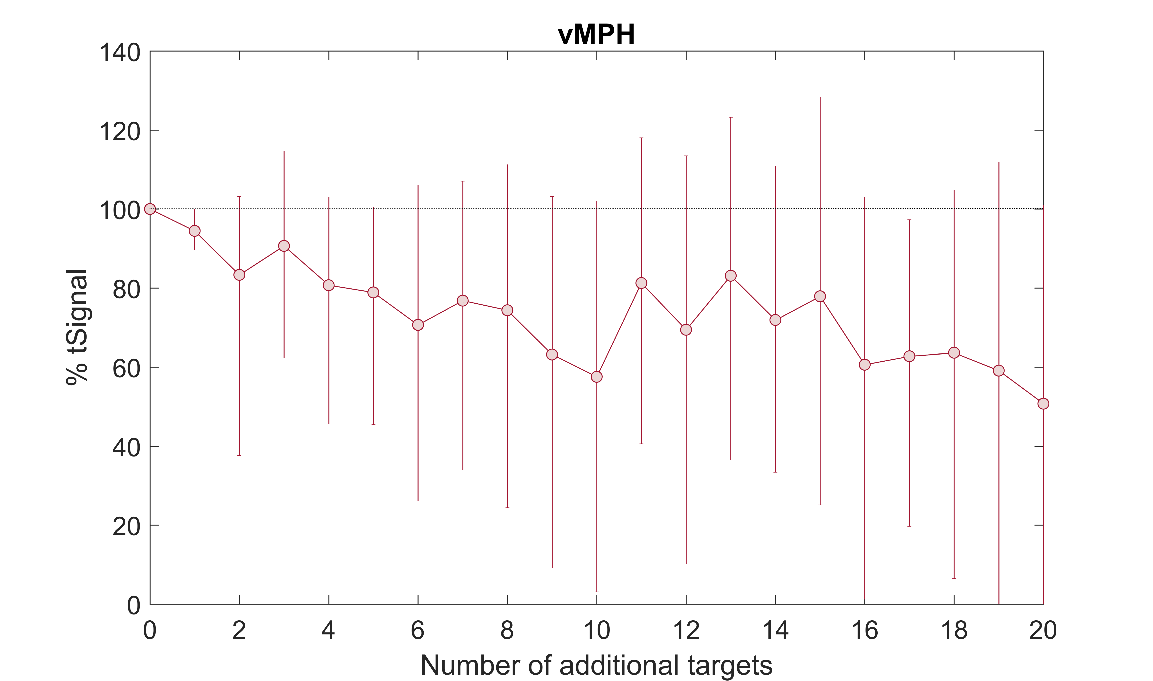


**Figure C. % conserved tSignal for extended target sets within the reference adult -ADHD TPMS models solutions.** The colored-dark lines and “o” markers correspond to the mean values of 50 random extra protein sets per each target addition, while vertical lines represent the upper and bottom standard deviations. The horizontal-black line delimitates the 100% tSignal. A) vLDX, B) vMPH. Similar results were obtained for the children-adolescent ADHD TPMS model solutions results (data not shown).

ADHD: Attention-deficit/hyperactivity disorder; TPMS: Therapeutic performance mapping system; vLDX: Virtual lisdexamfetamine; vMPH: Virtual methylphenidate.

| 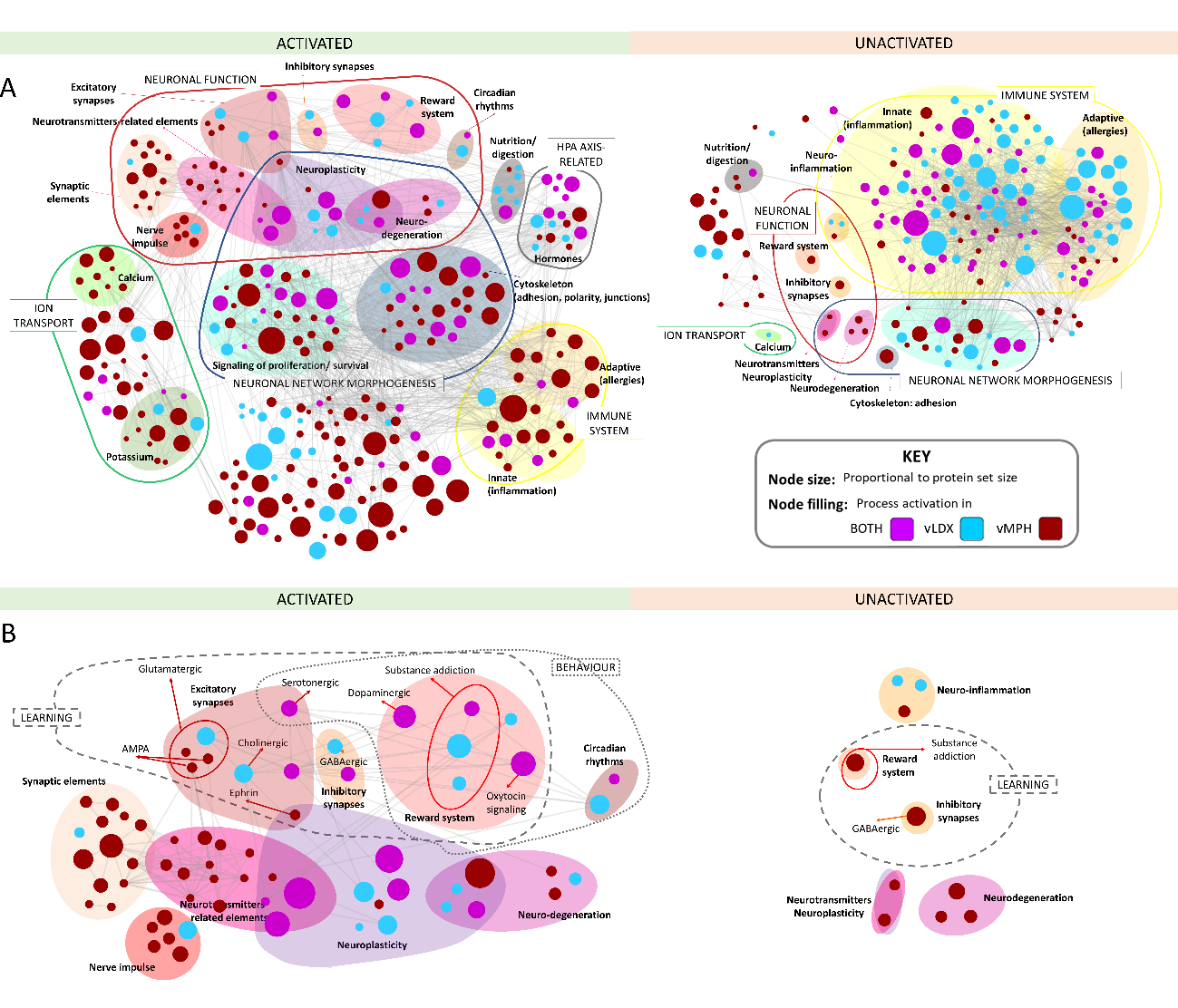 |
| --- |
| **Figure D. ADHD pathophysiology processes modulated (activated or inhibited) by vLDX and vMPH in the paediatric-adolescent population.** A) General overview. B) Neuronal-related processes.  ADHD: Attention-deficit/hyperactivity disorder; AMPA: γ-amino-3-hydroxy-5-methylisoxazole-4-propionic acid; vLDX: Virtual lisdexamfetamine; vMPH: Virtual methylphenidate |

| 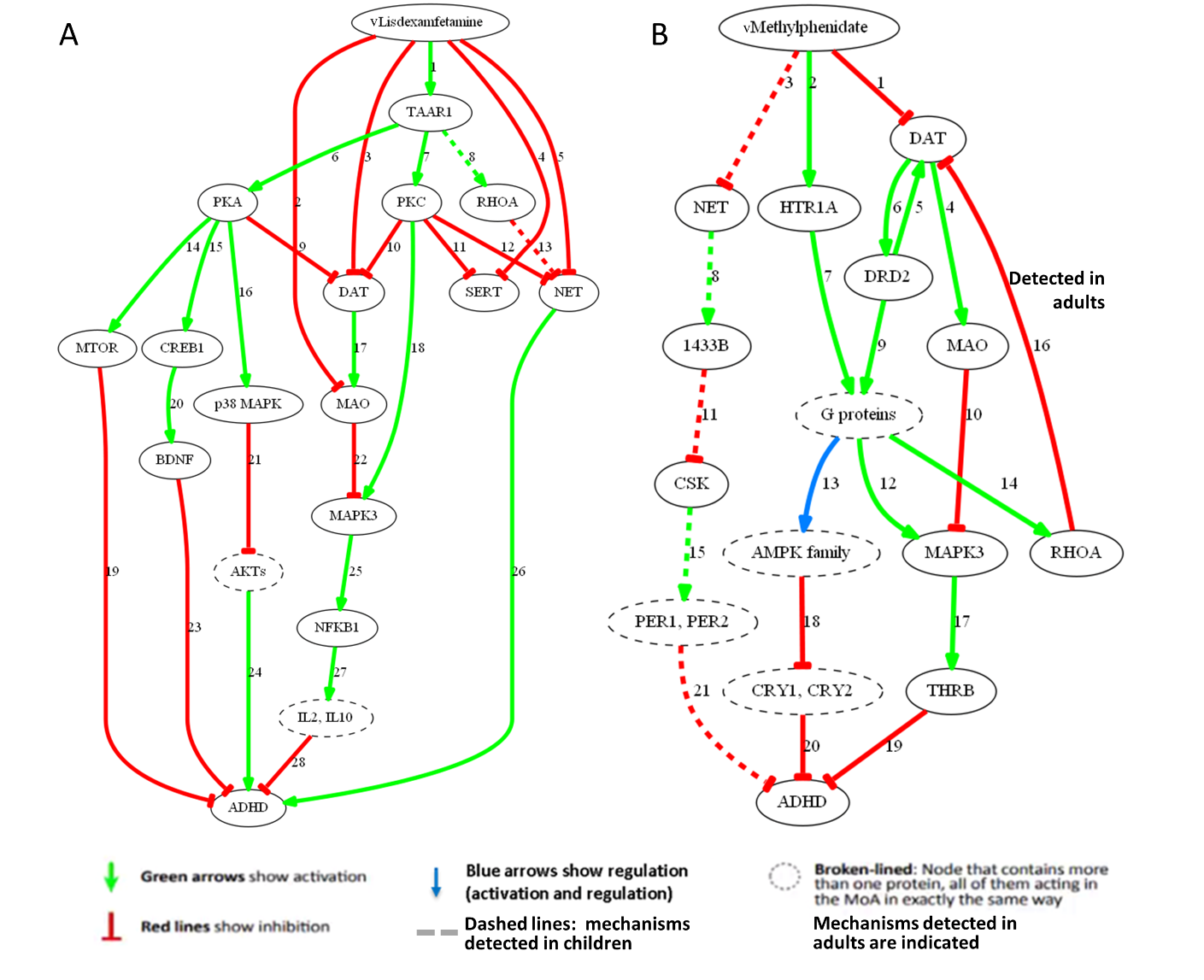 |
| --- |
| **Figure E. Detailed predicted mechanism of action of A) vLDX and B) vMPH in ADHD, with supporting bibliography.** Supplementary Table D contains the sources of information found in the scientific literature supporting the predicted mechanisms for vLDX and supplementary Table F for vMPH.  ADHD: Attention-deficit/hyperactivity disorder; vLDX: Virtual lisdexamfetamine; vMPH: Virtual methylphenidate |

| **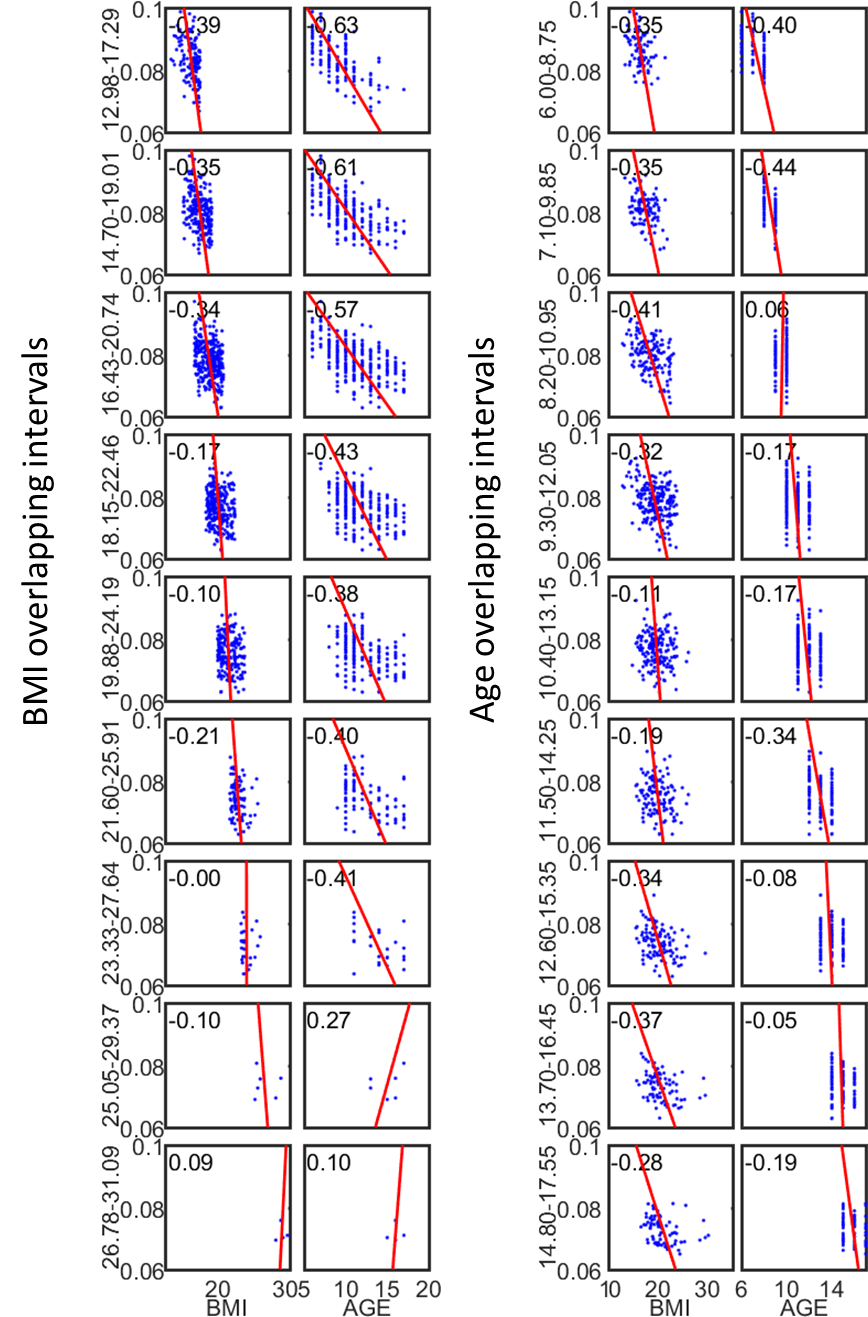** | |
| --- | --- |
| **Supplementary Figure F. Correlation between ADHD tSignal induced by vMPH and age or BMI when dividing the population in overlapping BMI or age intervals.** The numbers in the upper-left corner indicate Pearson’s correlation coefficient (ρ). Direction: positive correlation (ρ >0) meant the higher BMI/age, the higher the efficacy; negative correlation (ρ <0) meant the higher BMI/age, the lower the efficacy. Strength: Strong: \|ρ\| ≥0.8; Moderate: 0.8 >\|ρ\| ≥0.5; Low: 0.5> \|ρ\| ≥0.3; Negligible: \|ρ\| <0.3 (not shown [–]).ADHD: Attention-deficit/hyperactivity disorder; BMI: Body mass index; vMPH: Virtual methylphenidate | |
| 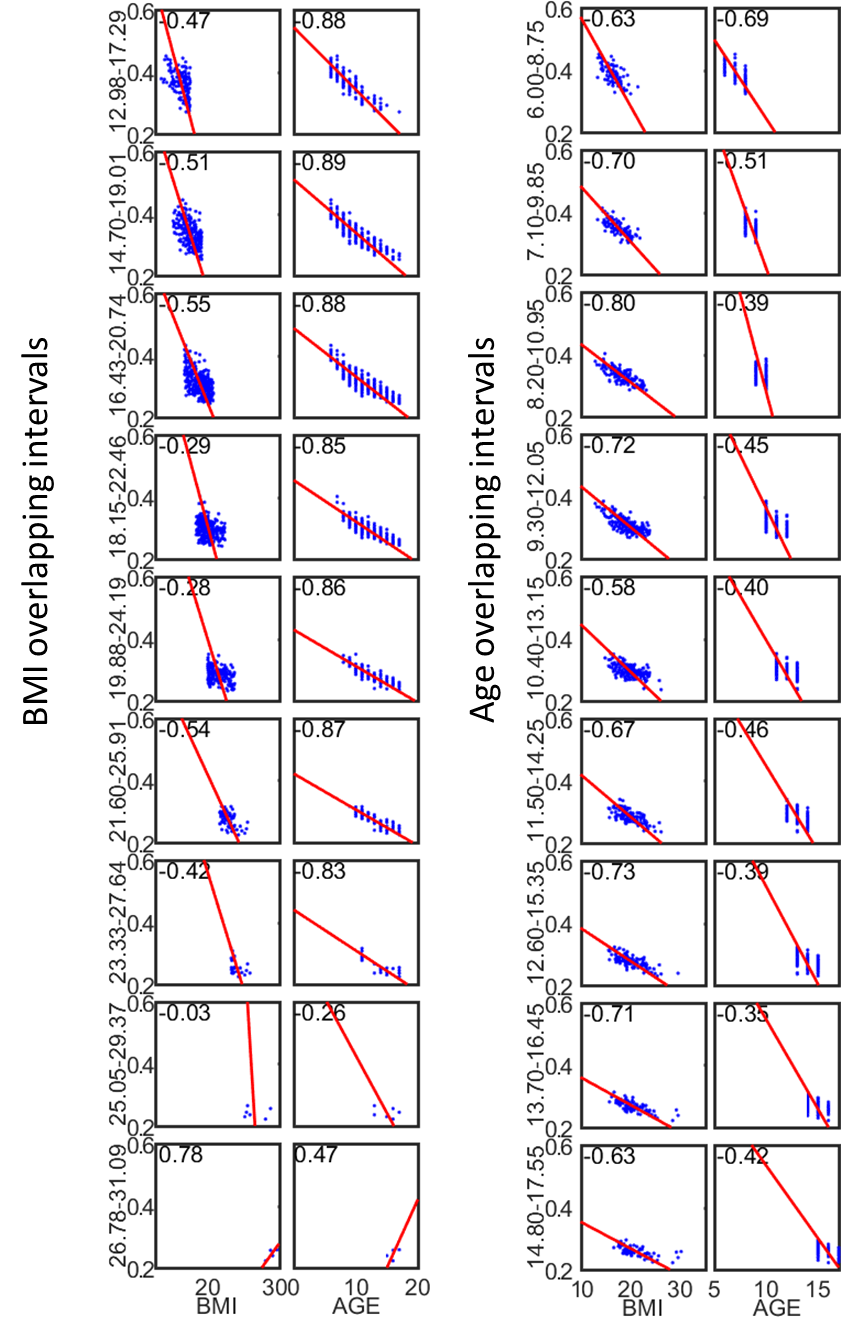 |  |
| **Supplementary Figure G. Correlation between ADHD tSignal induced by vLDX and age or BMI when dividing the population in overlapping BMI or age intervals.** The numbers in the upper-left corner indicate Pearson’s correlation coefficient (ρ). Direction: positive correlation (ρ >0) meant the higher the BMI/age, the higher the efficacy; negative correlation (ρ <0) meant the higher BMI/age, the lower the efficacy. Strength: Strong: \|ρ\| ≥0.8; Moderate: 0.8 >\|ρ\| ≥0.5; Low: 0.5> \|ρ\| ≥0.3; Negligible: \|ρ\| <0.3 (not shown [–]). ADHD: Attention-deficit/hyperactivity disorder; BMI: Body mass index; vLDX: Virtual lisdexamfetamine | |
